# Deciphering the underlying immune network of the potato defense response inhibition by *Phytophthora infestans* nuclear effector Pi07586 through transcriptome analysis

**DOI:** 10.3389/fpls.2023.1269959

**Published:** 2023-09-22

**Authors:** Yumeng Xiong, Di Zhao, Shengnan Chen, Lan Yuan, Die Zhang, Hongyang Wang

**Affiliations:** ^1^ Yunnan Key Laboratory of Potato Biology, Yunnan Normal University, Kunming, China; ^2^ School of Life Science, Yunnan Normal University, Kunming, China

**Keywords:** potato, late blight, *Phytophthora infestans*, effector, RNA-seq

## Abstract

Phytophthora infestans, a highly destructive plant oomycete pathogen, is responsible for causing late blight in potatoes worldwide. To successfully infect host cells and evade immunity, P. infestans secretes various effectors into host cells and exclusively targets the host nucleus. However, the precise mechanisms by which these effectors manipulate host gene expression and reprogram defenses remain poorly understood. In this study, we focused on a nuclear-targeted effector, Pi07586, which has been implicated in immune suppression. Quantitative real-time PCR (qRT-PCR) analysis showed Pi07586 was significant up-regulation during the early stages of infection. Agrobacterium-induced transient expression revealed that Pi07586 localized in the nucleus of leaf cells. Overexpression of Pi07586 resulted in increased leaf colonization by P. infestans. RNA-seq analysis revealed that Pi07586 effectively suppressed the expression of PR-1C-like and photosynthetic antenna protein genes. Furthermore, high-performance liquid chromatography-tandem mass spectrometry (HPLC-MS) analysis indicated that Pi07586 overexpression led to a substantial decrease in abscisic acid (ABA), jasmonic acid (JA), and jasmonoyl-isoleucine (JA-Ile) levels, while not affecting salicylic acid (SA) and indole-3-acetic acid (IAA) production. These findings shed new light on the modulation of plant immunity by Pi07586 and enhance our understanding of the intricate relationship between P. infestans and host plants.

## 1 Introduction

Potato (*Solanum tuberosum L.*) ranks as the third most crucial food crop, serving as a staple for 1.3 billion people worldwide (Stokstad, 2019). However, potato cultivation faces persistent challenges due to late blight, caused by *Phytophthora infestans*. This pathogen alone causes annual global economic losses of approximately $10 billion (Dong and Zhou, 2022). The ongoing battle between potato and *P. infestans* involves intricate and strategic confrontation strategies developed through long-term co-evolution. These strategies manifest in two branches of the immune system: basal or horizontal disease resistance and R (resistance) gene-based or vertical disease resistance (Boller and Felix, 2009). The first branch relies on plants’ transmembrane pattern recognition receptors (PRRs) to detect and respond to pathogen-associated molecular patterns (PAMPs). Examples include chitin for fungi, peptidoglycan for bacteria, and flagellin, which trigger the induction of PAMP-triggered immunity (PTI) and inhibit pathogen colonization. In the second mechanism, the intracellular immune receptors specifically recognize some cytoplasmic effectors to induce hypersensitive response (HR), termed effector-triggered immunity (ETI) (Jones and Dangl, 2006; Hein et al., 2009).

For successful colonization, adapted *Phytophthora* pathogens secrete two groups of effectors (apoplastic and cytoplasmic effectors) into plant cells to facilitate infection by suppressing host defense and physiological processes (Wang and Jiao, 2019). Apoplastic effectors are secreted into plant extracellular spaces and interact with extracellular defense-related factors. For example, *P. infestans* extracellular cystatin-like protease inhibitors EPIC1 and EPIC2B bind and inhibit the papain-like cysteine protease C14 (Kaschani et al., 2010). Kazal-like serine protease inhibitors, including the EPI1 and EPI10 proteins secreted by *P. infestans*, inhibit tomato subtilase P69B (Tian et al., 2004; Tian et al., 2005). In addition, some apoplastic effectors, such as NLPs and elicitins, could induce cell death. Until now, 61 cell death-inducing apoplastic effectors have been reported in 15 *Phytophthora* (Midgley et al., 2022). Cytoplasmic effectors are secreted into the cytoplasm via known and unknown mechanisms and are localized in various plant subcellular regions. The published genomic data indicate that *P. infestans* cytoplasmic effectors mainly produce two types, namely RxLR and CRN (Haas et al., 2009). RxLR effectors are modular architecture, including an N-terminal signal peptide for protein secretion, a conserved RxLR (Arg-any residue-Leu-Arg) motif to facilitate translocation into host cells, and a diverse C-terminal domain executing virulence activity (Morgan and Kamoun, 2007). Notably, these effectors exhibit sequence and expression polymorphisms between pathogen strains. Many recent functional studies have been conducted on RxLR effectors. For instance, PcAvh103, an avirulence homolog RxLR effector in *Phytophthora capsici*, targets host EDS1 to suppress plant immunity. Similarly, the RxLR effector Pi20303 evades recognition by the resistance protein Rpi-blb2, suppressing PTI and facilitating pathogen colonization through its interaction with and stabilization of StMKK1, a component of the potato MAPK cascade protein (Du et al., 2021). Another nucleus-localized RxLR effector, PsAvh110 in *Phytophthora sojae*, modulates the promoter activity of immune-associated genes by targeting the heterochromatin complex (Qiu et al., 2023). CRNs are also modular proteins that were first found in *P. infestans* and classified as genes causing crinkling and necrosis (Torto et al., 2003). These effector proteins possess a conserved LxLFLAK motif in N-terminal, which functions in the translocation of the CRN proteins from the apoplast into the plant cytoplasm (Schornack et al., 2010). In addition to inducing cell death in plants, such as CRN2 and CRN8 (van Damme et al., 2012), recent studies have shown that the majority of CRNs act in suppressing host defenses (Chen et al., 2013; Stam et al., 2013). Some *Phytophthora* spp. have been shown to have at least two CRNs with contradicting functions, one inhibiting cell death and the other inducing cell death (Midgley et al., 2022). For instance, CRN63 and CRN115 from *P. sojae*, CRN63 induces cell death, while CRN115 inhibits CRN63 induced cell death by interfering with catalases and inhibiting H_2_O_2_ accumulation (Zhang et al., 2015).

Within the *P. infestans* strain T30-4 genome, 563 RxLRs and 196 CRNs effectors have been identified (Haas et al., 2009). Are there any other types of cytoplasmic effectors besides RxLRs and CRNs that promote infection of the *P.infestans* secretome. A study indicated that 1415 secretome genes were predicted, including 563 apoplastic effectors and 852 cytoplasmic effectors. In addition to RxLR and CRN effectors, there are approximately 300 other unconventional types of cytoplasmic effectors (Raffaele et al., 2010). The effector *Pi07586* (Genbank no. XM_002904522.1, 447 bp) as a conserved hypothetical virulence effector gene was first reported in Raffaele et al., 2010. Unlike RxLR or CRN effectors, Pi07586 lacks an RxLR/LxLFLAK domain but contains a nuclear localization signal (NLS), and does not have significant similarities to known proteins and sequence motifs. Transcriptome data of potato/tomato infected by *P. infestans* indicates that *Pi07586* was up-regulated during the early stages of infection (Raffaele et al., 2010; Cooke et al., 2012). These suggest that Pi07586 is a new virulence protein different from RxLR or CRN effectors in *P. infestans*. However, knowledge of molecular mechanisms and how Pi07586 suppresses plant immunity is very limited.

Here, we identify and characterize that an effector Pi07586 localizes in the nucleus, and promotes *P. infestans* infection. Through the establishment of stable transgenic potato plants via genetic transformation, we identified 793 DEGs associated with reduced immune response using RNA-Seq. KEGG enrichment analysis revealed that downregulated genes, such as pathogenesis-related protein 1C-like and photosynthetic antenna protein, and upregulated genes, including ethylene-responsive transcription factor 1B-like and ethylene-responsive transcription factor ERF096-like, in Pi07586 transgenic plants, play crucial roles in affecting the sensitivity to *P. infestans*. Additionally, we assessed the phytohormone content of transgenic plants, revealing a correlation between their pathogen susceptibility and abscisic acid (ABA), jasmonic acid (JA), and jasmonoyl-isoleucine (JA-Ile). Collectively, our research sheds new light on the perturbation of host immune responses at the transcriptome level by the oomycete effector Pi07586, filling the existing gap in nuclear effector-related investigations.

## 2 Materials and methods

### 2.1 Plant material and microbial strains

Potato sterile seedlings of cultivar Désirée, *Nicotiana benthamiana* seeds, and the transient expression vector pRI101-GFP were provided by the Key Laboratory for Potato Biology of Yunnan province. Plants were grown under standard conditions (22–26°C; 16 h light/8 h dark photoperiod; and 70% relative humidity) in the laboratory greenhouse. Désirée and the *Pi07586* transgenic lines grow for ~5–6 weeks for *P. infestans* inoculation. The *N. benthamiana* grows ~4–5 weeks for transient expression. The *Agrobacterium* strain used for transient expression is GV3101 (Tsingke Biotechnology, Beijing, China). The *P. infestans* strain 88069 was propagated and stored in the rye V8 medium, and incubated at 18°C for 14 days.

### 2.2 *P. infestans* inoculation

The *P. infestans* strain 88069 grown for 14 days was taken and ddH_2_O was added to the culture dish, with the sporangia being scraped off the dish with an applicator. After filtration with microcloth, the final concentration of suspension was adjusted to 50 sporangia/μL. The suspension was stored at 4°C for 1 h. Then 10 μL suspension was placed on both sides of the detached potato leaf, using ddH_2_O as the control. After 24 h of dark humid cultivation under 16 h light/8 h dark conditions, the potato leaves were taken at 0, 24, 48, and 72 h post infection and stored at -80°C for later experiments. Three samples at each time point were repeated for a total of three biological replicates.

### 2.3 Quantitative real-time PCR analysis

RNA was extracted according to the plant RNA extraction kit (Omega, R6827-02, Guangzhou, China) as per the manufacturer’s instructions. Reverse transcription was performed using the TaRaKa reagent (TaKaRa, Beijing, China) to obtain cDNA. The qRT-PCR reaction was conducted according to TB Green Premix Ex Taq II (TaKaRa, Beijing, China). The *Pief2* (XM_002901697.1) gene of *P. infestans* was used as the internal reference gene. The 2^-ΔΔCt^ method was used to calculate the relative gene expression (Livak and Schmittgen, 2001). The qRT-PCR analysis of each sample was performed in triplicates.

### 2.4 Subcellular localization

*Agrobacterium tumefaciens* carrying the *p19*, *GFP-EV* (pRI101*-GFP*), and *GFP-Pi07586* (pRI101-*GFP-Pi07586*) recombinant plasmids were activated and cultured on LB solid medium with corresponding antibiotics at 28 °C for 2-3 days. The culture was then centrifuged at 3000 rpm for 10 min. A mixture of MMA (10 mM MES, 10 mM MgCl_2_, and 200 mM acetosyringone; pH = 5.6) was prepared to achieve an OD_600_ of 0.01. GFP-EV and GFP-Pi07586 were combined with p19 in equal volumes and incubated for 1 h. The bacterial solution was injected onto the surface of *N. benthamiana* leaves using a needleless 1 mL syringe. After 36-48 h of transient expression, the epidermis was carefully removed using tweezers and placed on a glass slide. Observation and photography were conducted using a laser confocal microscope.

### 2.5 Protein extraction and Western blot analysis

*N. benthamiana* plants were infiltrated with *A. tumefaciens* (OD_600_ = 0.3) containing the targeted genes. Leaves were harvested 48 h post-infiltration (hpi) and 100 mg of leaf tissue was placed in 1 mL of PBS lysis buffer with protease inhibitors. After grinding and centrifugation, the sample concentration was confirmed using the BCA protein content detection kit (Keygen Biotech, Nanjing, China). The protein sample was mixed with 1× SDS-PAGE loading buffer and boiled to denature the protein. The processed sample (20 μL) was loaded onto an SDS-PAGE gel (SmartPAGE™ Precast Protein Gel 12%, V522793, Changzhou, China) and electrophoretically separated in 1× MOPS SDS running buffer at 120 V for 1.5 h. One gel was transferred to a PVDF membrane at 240 mA for 1 h, while another gel was stained with Coomassie Blue Staining Solution (Beyotime Biotechnology, P0017, Shanghai, China) for loading visualization. The PVDF membrane was blocked with 5% milk in 1× TBST for 1 h to prevent non-specific binding. The primary antibody (ZEN-BIOSCIENCE, 300943, Chengdu, China) was diluted according to the instructions and incubated with the membrane. The membrane was washed thrice every 30 min with 1× TBST. Subsequently, the PVDF membrane was incubated with the secondary antibody (Bioss, bs-0295G-HRP, Beijing, China) at the recommended dilution in 1× TBST. The membrane was washed thrice every 10 min with 1× TBST. The Immobilion Western Chemiluminescence HRP Substrate color development kit (Millipore, WBKLS0100, Massachusetts, American) was added to the PVDF membrane, and a chemiluminescence instrument was used for luminescence photography.

### 2.6 Gene transformation of potato

The method was performed as described previously (Banerjee et al., 2006). The pRI101-*Pi07586* overexpression vector was successfully constructed and transformed into *Agrobacterium* GV3101. Stem segments of the potato cv. Désirée were used for genetic transformation. Explants were infected with *Agrobacterium* and cultured in the Z1N2AS medium (MS20 1L+ZT 1 mL+NAA (10 mg/mL) 200 μL+AS 1 mL) for two days in darkness. Subsequently, the explants were transferred to the recovery medium (MS20 1L+ZT 2 mL+NAA (0.1 mg/mL) 100 μL+TMT 2 mL) where they were dedifferentiated and formed calli. The calli were then transferred to the differentiation medium Z2N0.01 (MS20 1L+ZT 2mL+NAA (0.1 mg/mL) 100 μL+TMT 2 mL+Kana 2 mL) and cultivated in a light incubator. After approximately two months, the calli differentiated into buds, and regenerated seedlings were obtained. Once the regenerated seedlings reached 1-2 cm in size, they were cut from the callus and transferred to the rooting medium (MS30 1L+TMT 2 mL+Kana 1 mL) for initial screening. Positive selection of Pi07586 was determined using PCR. The culture medium formulas mentioned above are prepared in a 1 L ratio.

### 2.7 cDNA library construction and Illumina sequencing

Total RNA quality was assessed using the NanoDrop 2000 spectrophotometer and Agent2100/LabChip GX. Upon qualification, the library was constructed. Firstly, mRNA was enriched using magnetic beads containing Oligo (dT) and randomly fragmented using the Fragmentation Buffer. Then, the first and second cDNA strands were synthesized using mRNA as a template, followed by their purification. The purified double-stranded cDNA was subjected to end repair, A-tail addition, and sequencing connections. AMPure XP beads were used for fragment size selection. Finally, PCR enrichment generated a cDNA library. Following cDNA library quality inspection, the Illumina NovaSeq6000 sequencing platform was used for PE150 mode sequencing.

### 2.8 Determination of the phytohormones concentration

The sample was ground in liquid nitrogen until fully crushed, then accurately weighed and placed in a glass test tube. Then, 10 times the volume of acetonitrile solution was added along with 8 μL internal standard mother liquor. Overnight extraction was performed at 4°C, followed by centrifugation at 12000 g for 5 min, and the final supernatant was obtained. Subsequently, the extraction was repeated twice with the precipitate using 5 times the volume of acetonitrile solution, and then the resulting supernatants were combined. To this, 35 mg of C18 filler was added and shaken vigorously for 30 s, centrifuged at 10000 g for 5 min, and the supernatant was discarded. The residue was dried with nitrogen at a 400 μL methanol resolution rate, then passed through a 0.22 μM organic phase filter membrane, and finally stored in a -20°C refrigerator for machine testing. The hormone content was measured using HPLC-MS.

## 3 Results

### 3.1 Pi07586 is localized in nucleus and significantly upregulated in the early stage of pathogen infection

**Figure 1:**
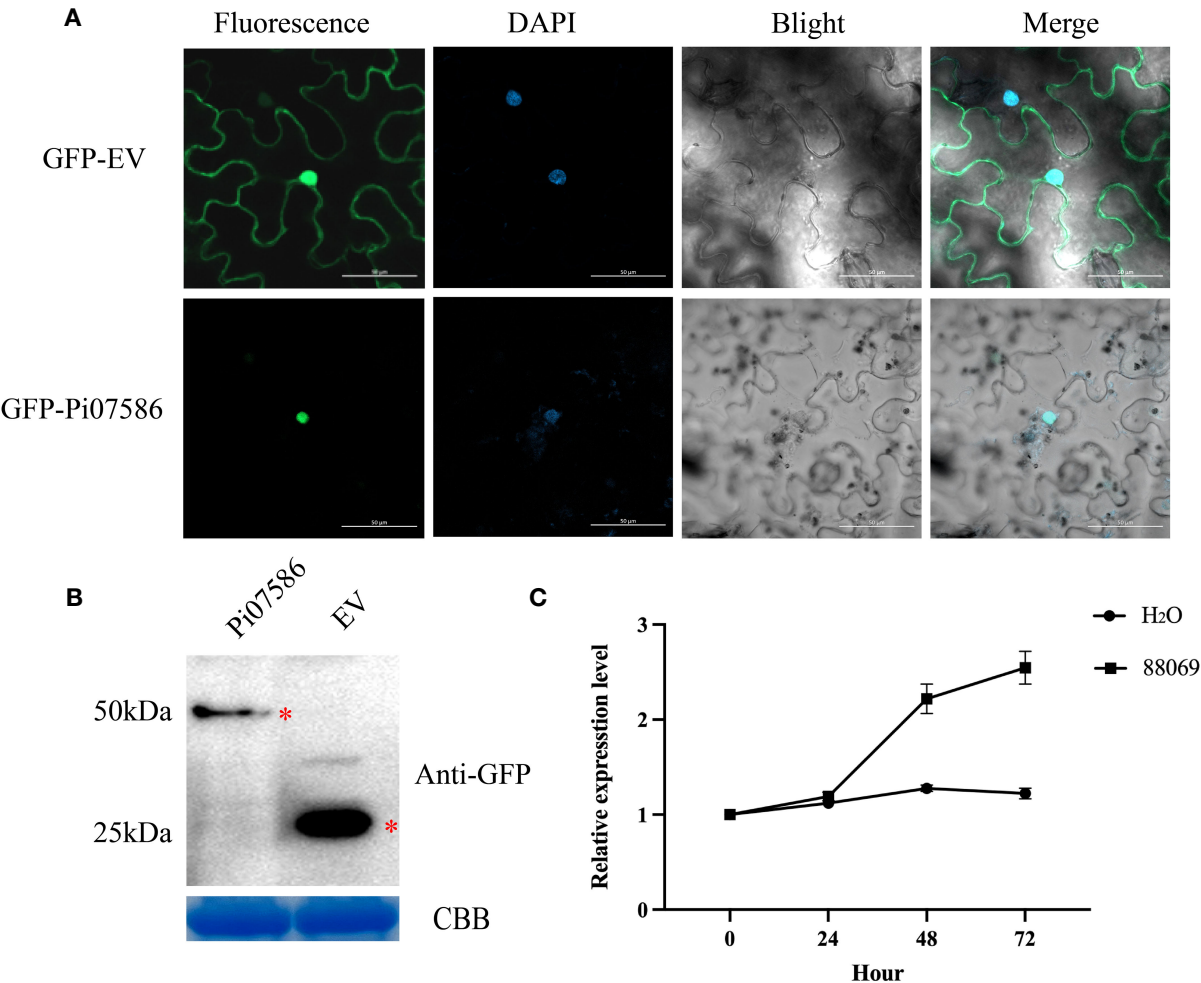
The localization and expression patterns of *Pi07586* during *P. infestans* infection. **(A)** Subcellular localization of Pi07586 in *Nicotiana benthamiana* epidermis cells was examined. The control group (GFP-EV) showed expression in the nucleus, cytoplasm, and cell membrane. The experimental group (GFP-Pi07586) exhibited expression specifically in the nucleus. Each panel displayed green fluorescence (left), DAPI nuclear dye (second), and merged images (third and extreme right). Scale bars represent 50 μm; **(B)** Western blot analysis confirmed Pi07586 expression. GFP-Pi07586 migrated at ~50 kDa, while GFP alone was detected as a 25 kDa band with anti-GFP antibodies. Coomassie blue (CBB) staining was used as the loading control; **(C)** Expression patterns of Pi07586 at different time points (0, 24, 48, and 72 h post-inoculation) were examined by qRT-PCR. H_2_O served as the control. Gene expression levels were calculated relative to the values at 0 h (control) using *Pief2* as the internal reference gene. Error bars represent means ± SDs from three independent experiments.

Previous transcriptome sequencing of *P. infestans* identified a candidate virulence effector gene, *Pi07586*, with a length of 447 bp, encoding a 148-amino acid protein lacking an RxLR/LxLFLAK domain but containing an NLS motif (**Supplementary Figure 1**). To determine the subcellular localization of Pi07586 and its functional role, the Pi07586 sequence, excluding the signal peptide, was fused with green fluorescent protein (GFP) under the control of the pRI101 vector. *Agrobacterium*-induced transient expression was performed on *N. benthamina* leaves. The results revealed that GFP-fused Pi07586 localized specifically in the nucleus of leaf cells (**Figure 1A**). Confocal microscopy imaging of DAPI-stained leaves confirmed the colocalization of GFP and the nuclei. Western blot analysis using a GFP-specific antibody demonstrated the stability of GFP-Pi07586 and empty vector fusion proteins, showing bands of the expected sizes, 50 kDa and 25 kDa, respectively (**Figure 1B**). To further investigate Pi07586 expression during potato infestation, potato leaves were infected with a weakly virulent *P. infestans* strain 88069. The expression of Pi07586 was analyzed at four time points (0, 24, 48, 72 h) post-infestation, with water-treated leaves as controls. qRT-PCR analysis (**Figure 1C**) revealed continuous upregulation of Pi07586 expression up to 72 h compared to the mock-infected control. These findings demonstrate the active involvement of Pi07586 in the induction of pathogenic infestation in potatoes.

### 3.2 Overexpression of Pi07586 in potatoes enhances *P. infestans* colonization

**Figure 2:**
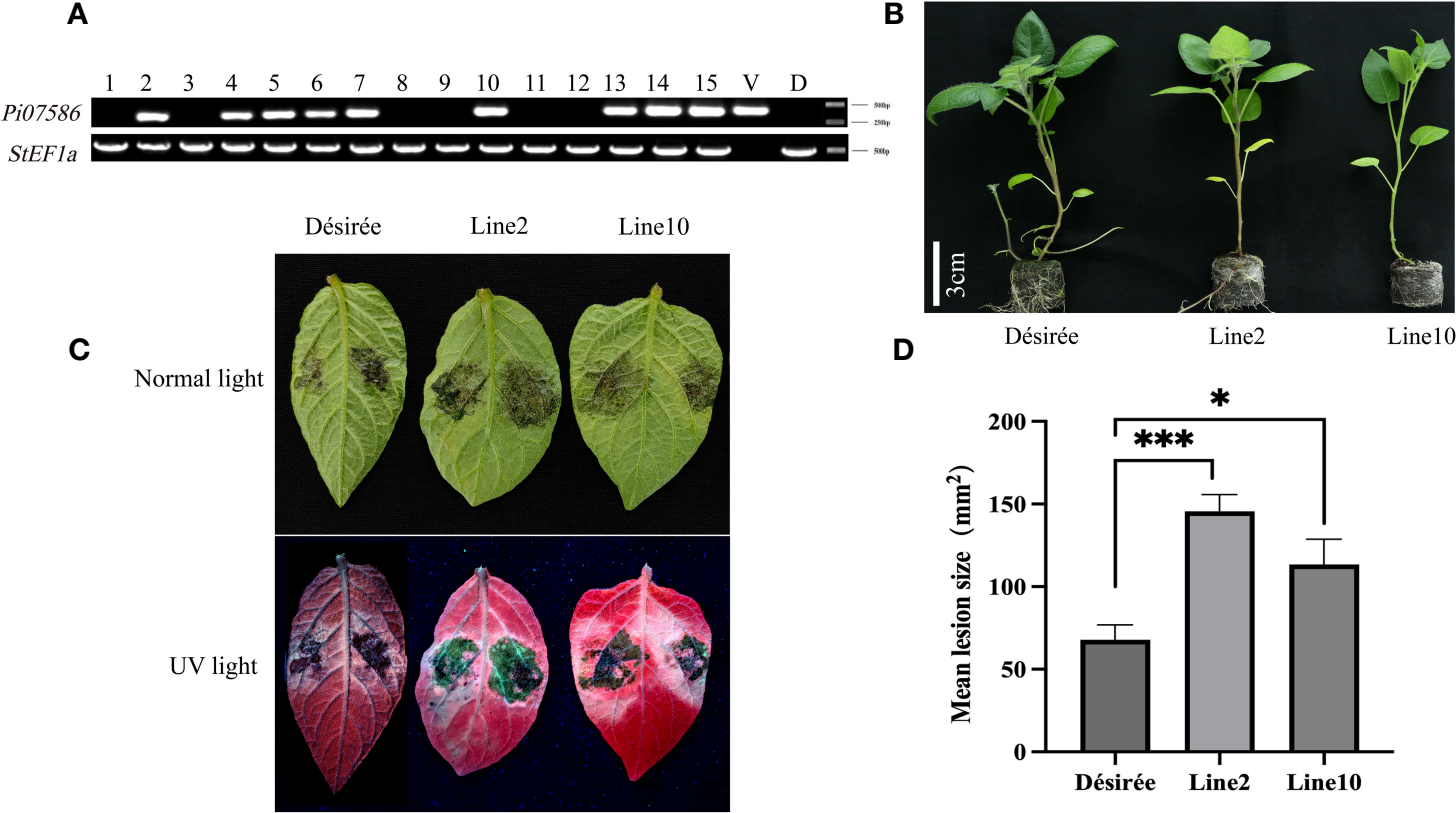
The susceptibility of *Pi07586* transgenic plants to *P. infestans* was higher compared to the Désirée. **(A)** PCR analysis identified nine positive potatoes among the 15 transgenic potatoes, using *StEF1α* as the internal reference gene. V (Vector) was a positive control. D (Désirée) was a negative control; **(B)** Height measurements were taken for Désirée, line 2 and line 10 (after three weeks of growth for sterile seedlings). Scale bars indicate 3 cm; **(C)** Four days after inoculating potato leaves with a suspension of *P. infestans* under normal and UV light, a picture was taken. The suspension count was 50 sporangium/μL; **(D)** The mean lesion size of detached potato leaves was significantly larger in transgenic potato lines (lines 2 and 10) compared to Désirée (one-way ANOVA; *, p < 0.05; ***, p < 0.001). Error bars represent SD from three independent experiments.

To investigate the virulence function of Pi07586 during *P. infestans* infection, we generated transgenic potato lines by introducing Pi07586 into the potato cultivar Désirée using *Agrobacterium*-mediated transformation. Two stable and high-level expression transgenic lines, line 2 and line 10, were selected for the *P. infestans* inoculation assay (**Figure 2A**, **Supplementary Figure 2**). The height, tubers’ number, and weight of Désirée, line 2, and line 10 did not show any significant differences (**Figure 2B**, **Supplementary Figures 3A, B**). We inoculated detached leaves from the transgenic lines and Désirée with *P. infestans* strain 88069. At four days post-inoculation (dpi), we observed larger disease lesions on the leaves of Pi07586 transgenic plants compared to Désirée (**Figures 2C, D**). These results clearly indicate that the Pi07586 transgenic lines exhibited increased susceptibility to *P. infestans* infection.

### 3.3 Identification of 793 differentially expressed genes throughout the RNA-seq analysis

**Figure 3:**
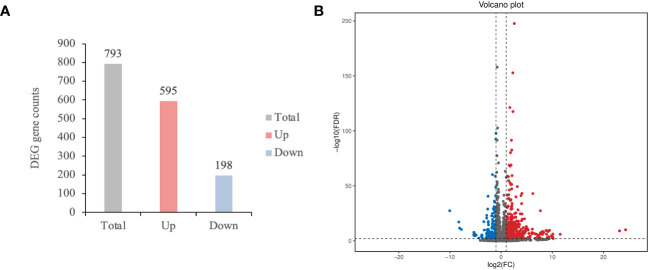
The number of differentially expressed genes (DEGs) and differential expression volcano plot. **(A)** The total, up and down-regulated DEGs were marked in grey, red, and blue color, respectively; **(B)** Screening DEGs based on Fold Change ≥ 2 and FDR < 0.01. Log2(FC) represents the logarithmic value of the different multiple. Each point represents a gene. The blue and dots represented the downregulated and upregulated DEGs, respectively, while the gray dots represented the non-differentially expressed genes.

Furthermore, we investigated the mechanisms of Pi07586 in potatoes. RNA-seq analysis was conducted on three biological replicates of Désirée, lines 2, and 10, 24 h of inoculation with the pathogen, with a total of nine samples The Illumina NovaSeq6000 sequencing platform was utilized for PE150 pattern sequencing. A total of 56.06 Gb of Clean Data was obtained, with each sample yielding 5.72 Gb of Clean Data, and a Q30 base percentage exceeding 91.70% (**Supplementary Table 1**). The RNA-seq readings for each library ranged from 86.71% to 88.11% and were successfully mapped to the potato genome (**Supplementary Table 2**). The Clean Reads of each sample were aligned against the *S. tuberosum* DM reference genome (v6.1) (Pham et al., 2020) using DESeq2 (Love et al., 2014) for differential analysis. Differential gene screening was performed with criteria of Fold Change ≥ 2 and FDR < 0.01, and the resulting differentially expressed gene (DEG) list underwent functional enrichment analysis (**Supplementary Table 3**). A total of 793 genes were identified, including 595 upregulated genes and 198 downregulated genes (**Figure 3A**). The volcano plot visually displayed the significance and variation in expression levels of the DEGs (**Figure 3B**). Among the upregulated genes, the gene with the largest log_2_(FC) was Soltu.DM.04G006870.2, encoding a transcription factor jumonji (jmj) family protein in the wild-type and transgenic plant comparisons. The second largest log_2_(FC) belonged to Soltu.DM.03G025990.1, which encodes a P-loop containing nucleoside triphosphate hydrolases superfamily protein. Notably, the downregulated gene with the largest fold change was a newly identified gene.

### 3.4 Gene ontology functional enrichment analysis of the DEGs

To understand the primary biological functions of the DEGs triggered by *Pi07586*, we conducted GO to examine the transcriptome changes. GO analysis encompasses three main branches: biological process, molecular function, and cellular component. Subsequently, we assigned DEGs to specific functional terms by annotating and classifying them at the GO database’s secondary classification level, thus providing insights into their primary functions. **Tables 1** and **2** present the DEGs with q-values of < 0.01. Pi07586 upregulates diverse types of DEGs involved in biological processes, including metabolic processes and immune system processes (**Table 1**). Among metabolic processes, upregulated DEGs primarily include carbohydrate metabolism (GO:0005975) and chitin catabolism (GO:0006032). Notably, carbohydrate metabolism exhibits the highest number of upregulated DEGs. Moreover, numerous DEGs associated with immune responses, such as defense response (GO:0006952) and innate immune response (GO:0045087), are enriched and upregulated in biological processes. This suggests that Pi07586 may influence plant sensitivity to pathogens by upregulating DEGs involved in metabolism and immune processes. Regarding cellular components, only the integral component of the membrane (GO:0016021) shows enrichment, comprising 164 upregulated DEGs. The three most enriched upregulated DEGs in molecular functions are heme binding (GO:0020037), oxidoreductase activity (GO:0016491), and UDP-glycosyltransferase activity (GO:0008194). Molecular functions also involve DEGs related to chitin, such as chitin binding (GO:0008061) and chitinase activity (GO:0004568), with 11 and 9 DEGs, respectively.

**Table 1:** GO enrichments of upregulated genes.

Term Type	ID	Description	GeneRatio	Enrich factor	qvalue	Gene number
Biological Process	GO:0005975	carbohydrate metabolic process	13.66%	3.75	1.13E-06	25
	GO:0006952	defense response	11.48%	2.55	0.00197265	21
	GO:0048544	recognition of pollen	8.74%	9.44	2.28E-09	16
	GO:0006979	response to oxidative stress	7.10%	3.18	0.00438241	13
	GO:0009607	response to biotic stimulus	6.56%	4.59	0.00034695	12
	GO:0050832	defense response to fungus	5.46%	8.42	1.39E-05	10
	GO:0042742	defense response to bacterium	5.46%	7.65	3.06E-05	10
	GO:0016998	cell wall macromolecule catabolic process	4.92%	29.32	2.28E-09	9
	GO:0006032	chitin catabolic process	4.92%	26.98	2.42E-09	9
	GO:0009814	defense response, incompatible interaction	4.37%	12.75	9.63E-06	8
	GO:0045087	innate immune response	4.37%	9.22	8.82E-05	8
	GO:0006955	immune response	4.37%	8.95	0.00010125	8
	GO:0002376	immune system process	4.37%	7.49	0.00032855	8
	GO:0006970	response to osmotic stress	4.37%	5.4	0.0025549	8
	GO:0098542	defense response to other organism	4.37%	5.21	0.00308632	8
	GO:0009651	response to salt stress	4.37%	4.95	0.00416784	8
	GO:0009817	defense response to fungus, incompatible interaction	3.83%	18.73	4.86E-06	7
	GO:0009620	response to fungus	3.83%	6.48	0.00246166	7
	GO:0009617	response to bacterium	3.83%	6.32	0.0025549	7
	GO:0009816	defense response to bacterium, incompatible interaction	3.28%	23.66	7.89E-06	6
	GO:0015706	nitrate transport	1.64%	56.2	0.00029895	3
Cellular Component	GO:0016021	integral component of membrane	71.49%	1.53	2.00E-12	163
Molecular Function	GO:0020037	heme binding	7.79%	2.16	0.00181145	30
	GO:0016491	oxidoreductase activity	7.01%	2.14	0.00350262	27
	GO:0008194	UDP-glycosyltransferase activity	4.68%	3.17	0.00057432	18
	GO:0008061	chitin binding	2.86%	12.94	4.95E-08	11
	GO:0004601	peroxidase activity	2.86%	3.57	0.00406964	11
	GO:0004568	chitinase activity	2.34%	22.45	1.58E-08	9
	GO:0016831	carboxy-lyase activity	1.82%	9.7	0.00035043	7
	GO:0016717	oxidoreductase activity	1.30%	10.75	0.00193553	5
	GO:0015276	ligand-gated ion channel activity	1.30%	8.43	0.00406964	5
	GO:0015204	urea transmembrane transporter activity	0.78%	46.77	0.00057432	3
	GO:0042973	glucan endo-1,3-beta-D-glucosidase activity	0.78%	23.38	0.00363566	3

**Table 2:** GO enrichments of downregulated genes.

Term Type	ID	Description	GeneRatio	Enrich factor	qvalue	Gene number
Biological Process	GO:0009765	photosynthesis, light harvesting	20.59%	57.62	1.10E-19	14
	GO:0018298	protein-chromophore linkage	20.59%	44.11	3.68E-18	14
	GO:0009611	response to wounding	7.35%	10.29	0.00581679	5
Cellular Component	GO:0016021	integral component of membrane	67.65%	1.45	0.00432162	46
	GO:0009523	photosystem II	22.06%	55.03	4.11E-21	15
	GO:0009535	chloroplast thylakoid membrane	22.06%	19.76	1.43E-14	15
	GO:0009522	photosystem I	20.59%	57.56	3.07E-20	14
	GO:0005615	extracellular space	4.41%	39.75	0.0007673	3
	GO:0009538	photosystem I reaction center	2.94%	43.36	0.00864725	2
Molecular Function	GO:0016168	chlorophyll binding	11.20%	45.57	3.92E-18	14
	GO:0004867	serine-type endopeptidase inhibitor activity	4%	19.6	0.00019764	5
	GO:0004869	cysteine-type endopeptidase inhibitor activity	3.20%	20.22	0.00105972	4

In contrast, GO enrichment analysis of downregulated DEGs reveals enrichment in photosynthetic-related processes, cellular components, and molecular functions (**Table 2**). These include photosynthesis and light harvesting (GO:0009765), photosystem II (GO:0009523), photosystem I (GO:0009522), photosystem I reaction center (GO:0009538), and chlorophyll binding (GO:0016168). The downregulation of these photosynthesis-related genes suggests that Pi07586 transgenic plants are more susceptible to the disease, potentially due to reduced plant resistance resulting from decreased photosynthesis. Interestingly, the downregulated cellular component also shows enrichment in 46 DEGs related to the integral component of the membrane (GO:0016021), suggesting their indirect relevance to the pathogenic function of Pi07586.

### 3.5 KEGG pathway enrichment analysis of the DEGs

**Figure 4:**
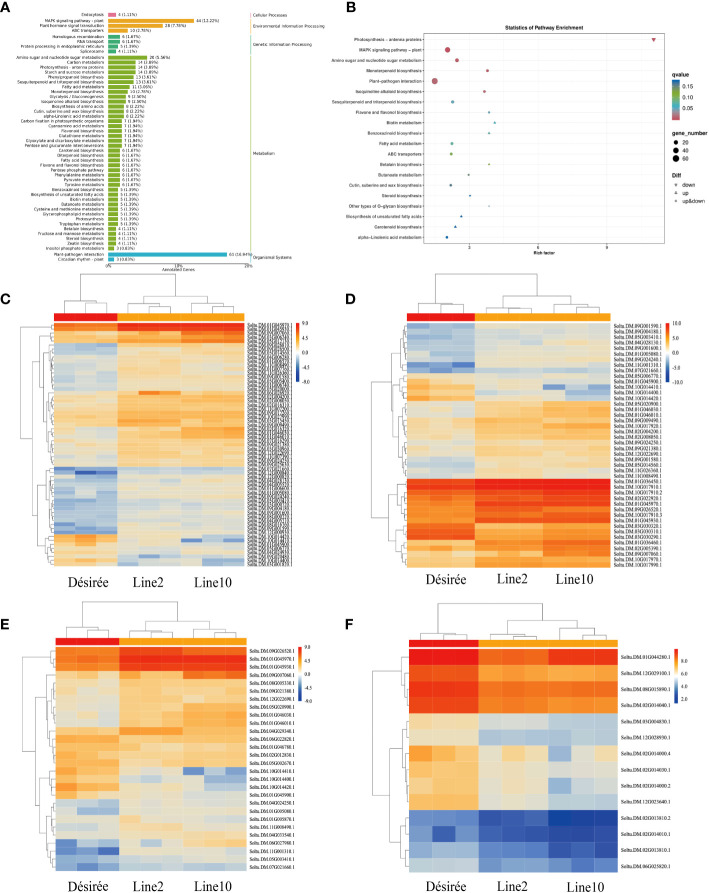
KEGG pathway enrichment of DEGs and DEGs gene expression was analyzed by qRT-PCR. **(A)** The bar plot illustrates the enrichment of KEGG pathways. The left vertical axis displays the annotated pathway names, while the right vertical axis shows the corresponding primary classification names. The horizontal axis represents the number of genes annotated to each pathway, relative to the total number of annotated genes; **(B)** The dot plot represents the KEGG pathway enrichment. The horizontal axis indicates the rich factor, while the vertical axis displays the pathway annotations. Upregulated genes are denoted by positive triangles, downregulated genes by inverted triangles, and both by circles. The color of each point corresponds to the q-value obtained from the hypergeometric test; **(C)** Heat map depicting the expression pattern of plant-pathogen interaction-related DEGs; **(D)** Heat map illustrating the expression pattern of MAPK signaling pathway-related DEGs; **(E)** Heat map displaying the expression pattern of plant hormone signal transduction-related DEGs; **(F)** Heat map showcasing the expression pattern of photosynthetic antenna protein-related DEGs.

To comprehend the biochemical metabolic and signal transduction pathways linked to DEGs, we performed KEGG annotation and functional classification analysis on 793 DEGs obtained from wild-type and transgenic lines. DEG annotation results were categorized into five types of KEGG pathways: cellular processes, environmental information processing, genetic information processing, metabolism, and organismal systems (**Figure 4A**). The most enriched genes belonged to the plant-pathogen interactions category within organismal systems, accounting for 16.94% (61 genes) of the total DEGs. Among them, 53 DEGs were upregulated and 8 DEGs were downregulated. Notably, three of the downregulated genes were predicted as pathogenesis-related protein 1C-like (PR-1C-like) (Soltu.DM.10G014410.1, Soltu.DM.10G014420.1, and Soltu.DM.10G014400.1). In environmental information processing, 44 and 28 genes were enriched in the MAPK signaling pathway and plant hormone signal transduction, respectively. Among the 44 DEGs enriched in the MAPK signaling pathway, 36 were upregulated and 8 were downregulated. Interestingly, three of the downregulated genes were also predicted as PR-1C-like, similar to those enriched in plant-pathogen interactions. Additionally, ethylene-responsive transcription factor 1B-like (ERF1B-like) (Soltu.DM.09G026520.1, Soltu.DM.11G001310.1, and Soltu.DM.05G020900.1) was enriched and upregulated in both the MAPK signaling pathway and plant hormone signal transduction. Similarly, ethylene-responsive transcription factor ERF096-like (ERF096-like) (Soltu.DM.09G026500.1) was enriched and upregulated in the plant-pathogen interactions pathway. Moreover, 14 downregulated genes related to photosynthetic antenna protein were enriched in metabolism (**Figure 4B**). The KEGG analysis revealed that i07586 primarily affects plant immunity through the plant-pathogen interactions pathway, MAPK signaling pathway, plant hormone signal transduction, and photosynthetic antenna protein (**Figures 4C–F**).

### 3.6 Validation of RNA-seq data by qRT-PCR

**Figure 5:**
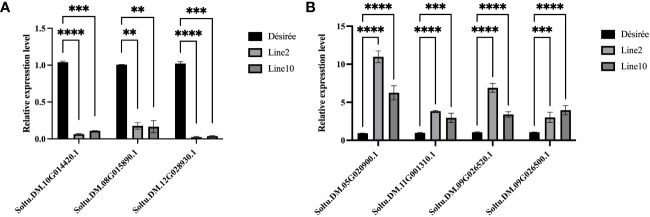
Validating the DEGs by qRT-PCR. **(A)** Three of the downregulated DEGs; **(B)** Four of the upregulated DEGs. The horizontal axis is the gene name. The internal reference gene is *StEF1α*. The error bars indicate means ± SDs from three independent experiments. One-way ANOVA, ns represents Not Statistically Significant; **, p < 0.01; ***, p < 0.0001; ****, p < 0.00001.

To demonstrate the accuracy of RNA-seq results, we identified several DEGs for qRT-PCR analysis. The relative expression levels of PR-1C-like (Soltu.DM.10G014420.1) and photosynthetic antenna protein (Soltu.DM.08G015890.1; Soltu.DM.12G028930.1) showed a significant decrease compared to Désirée (**Figure 5A**). Conversely, the expression levels of ERF1B-like (Soltu.DM.05G020900.1; Soltu.DM.11G001310.1; Soltu.DM.09G026520.1) and ERF096-like (Soltu.DM.09G026 500.1) displayed a significant increase compared to Désirée (**Figure 5B**). These results indicate that Pi07586 hampers plant resistance by modulating the plant immune network, primarily by downregulating genes associated with PR-1C-like and photosynthetic antenna protein, while upregulating genes linked to ERF1B-like and ERF096-like.

### 3.7 Pi07586 affects disease resistance by regulating phytohormones content

**Figure 6:**
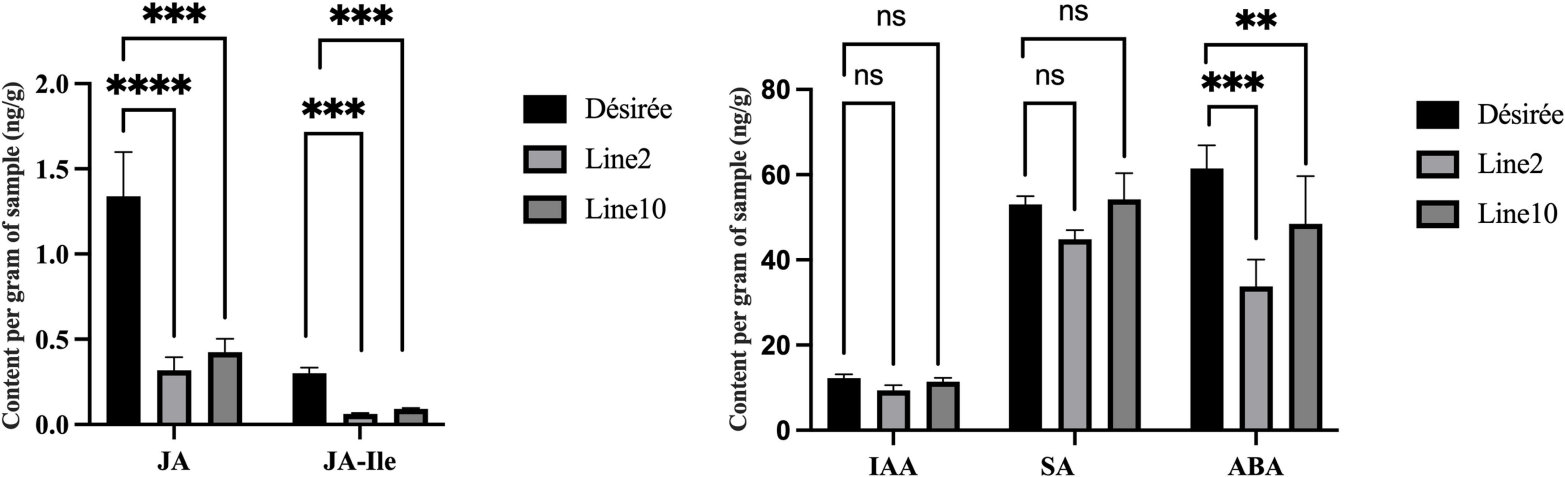
The endogenous hormones content of transgenic plants and wild type. Detection of hormone content (JA, IAA, JA-Ile, ABA, and SA) in detached leaves inoculated with late blight pathogen 88069. The error bars indicate means ± SDs from three sample repetitions. One-way ANOVA, ns represents Not Statistically Significant; **, p < 0.01; ***, p < 0.0001; ****, p < 0.00001.

Endogenous plant hormones, synthesized as small molecule compounds, act as signaling substances in response to pathogen attacks. These include JA, JA-Ile, IAA, ABA, and SA, which activate disease-resistance genes and enhance plant defense. Therefore, to quantify JA, IAA, JA–Ile, ABA, and SA, we used high-performance liquid chromatography-tandem mass spectrometry (HPLC-MS) for both Désirée and transgenic 2 and 10 lines. Interestingly, transgenic lines showed reduced ABA, JA, and JA-Ile compared to Désirée (**Figure 6**), while IAA and SA level changes remained nonsignificant. Lower JA and JA-Ile contents reduce the potato resistance to *P. infestans*, with ABA reduction possibly facilitating *P. infestans* colonization in the necrotrophic stage.

## 4 Discussion

*P. infestans* successfully causes late blight on tomatoes and potatoes, which is inseparable from a large number of apoplastic and cytoplasmic effectors. Apoplastic effectors accumulate in the intercellular spaces and interact with extracellular defense-related factors, whereas cytoplasmic effectors are secreted into intercellular spaces of host cells and are recognized by host plants to activate immunity or act as a virulence factor to suppress plant immunity (Sharpee and Dean, 2016). Compared with the most well-studied classes of *Phytophthora* cytoplasmic effectors, such as CRNs and RxLRs, our understanding of other types of cytoplasmic effectors is limited (Midgley et al., 2022). Recent studies suggest that, like RxLR effectors, some conserved hypothetical proteins are also upregulated at the early infection stage (Raffaele et al., 2010; Cooke et al., 2012). However, the functions of conserved hypothetical proteins are largely unknown. In this study, we identified that a conserved hypothetical protein encoded by *Pi07586* is upregulated during the infection stage of *P. infestans*. Through the establishment of stable transgenic potatoes, we proved that *Pi07586* promoted the susceptibility of potatoes to *P. infestans*.

The nucleus is the active center of PTI and ETI, and many critical regulators are trafficked there from various subcellular locations following pathogen perception (Wang et al., 2019b). Previous research has identified several virulence RxLR effectors expressed in the nucleus during infection, including Pi07550, Pi06094, Pi04314, Pi22798, and Pi16294 (Wang et al., 2019b). And nuclear localization signal of effector PsCRN108 was required for its contribution to virulence and its suppression of plant *HSP* gene expression (Song et al., 2015). In our study, the subcellular localization was confirmed through transient expression in *N. benthamiana* and western blot confirmed the stable expression of Pi07586 in the nucleus (**Figures 1A, B**). These results are consistent with previous reports (Zhang et al., 2021) and suggest that Pi07586 may implement its function in the nucleus. As a virulence factor, some *Phytophthora* effectors play a role in pathogen infection at various stages, with *Pi22798* being found to be upregulated at 24 h and 48 h after inoculation of potato plants with *P. infestans* (Wang et al., 2017). And the apoplastic effectors encoded by *NEPs* from *P. infestans*, *P. megakarya*, and *P. capsica* also are upregulated in the early infection stage (Bae et al., 2005; Haas et al., 2009; Chen et al., 2013). In our study, we used qRT-PCR to demonstrate continuous upregulation of *Pi07586* in the early stages of infection, from 24 h to 72 h (**Figure 1C**). These findings suggest that Pi07586 effectively regulates plant immunity and promotes pathogen infection in plants.

RNA-Seq has been a valuable method for transcriptome dynamics analysis in the interaction between potatoes and their pathogen (Li et al., 2022). For example, one study identified 190 differentially expressed genes (DEGs) in *Pi15718.2* transgenic potato lines and investigated the relationship between these DEGs, immune suppression, and plant growth (Wang et al., 2019a). Similarly, RNA-Seq analysis of *Pi04089* transgenic lines revealed 658 upregulated genes and 722 downregulated genes, with *Pi04089* significantly suppressing flg22-triggered defense signaling in potato plants (Luo et al., 2021). In this study, RNA-Seq analysis has been employed in studying the functions of effectors. In total, 793 DEGs were identified in the *Pi07586* transgenic lines, including 595 upregulated and 198 downregulated (**Figure 3**). Go enrichment analysis indicated that DEGs in *Pi07586* transgenic potato plants were focused around 43 GO terms (**Table 1**, **2**). KEGG pathway enrichment of these DEGs revealed the top five enriched pathways are photosynthesis-antenna proteins, MAPK signaling pathway-plant, amino sugar and nucleotide sugar metabolism, monoterpenoid biosynthesis, and plant-pathogen interaction (**Figure 4**). Many genes are related to plant immunity, which may help to explain the virulence function of Pi07586.

Plant chitinases, belonging to glycosyl hydrolase family 19, are predominantly endochitinases. These enzymes cleave chitin randomly, generating various chitooligosaccharides (Iseli et al., 1996). Previous studies have demonstrated the involvement of plant chitinases in defense responses, triggering the release of pathogenesis response proteins upon pathogenic attack (Vaghela et al., 2022; Chouhan et al., 2023). In this study, 8 out of 16 up-regulated genes are chitinase family genes in the amino sugar and nucleotide sugar metabolism pathway (**Supplementary Table 3**). These data are somewhat inconsistent with the toxic function of Pi07586. We speculate that it may be a plant’s common response to receive foreign substances. In addition, 3 out of the 4 down-regulated genes were chitin-binding lectins that reduced expression levels by 2.5 times. Some studies showed that chitin-binding lectins have anti-fungal and insecticidal activities (Chen et al., 2018). It is worth investigating whether chitin-binding lectins are involved in Pi07586-mediated plant immunity.

Ethylene (ET) plays a vital role in plant developmental processes and immune responses to pathogen attacks. Previous research has shown that ethylene response factors (ERFs) can positively or negatively regulate plant resistance. For instance, AtERF1, an upstream component of JA and ET signaling, contributes to pathogen resistance (Cheng et al., 2013). Transgenic *Arabidopsis* plants overexpressing *VpERF1* show enhanced susceptibility to the fungal pathogen *P. parasitica* var. *nicotianae* Tucker (Zhu et al., 2013). Similarly, *ERF5* negatively regulates the plant signaling and defense against the fungal pathogen *Alternaria brassicicola* (Son et al., 2012). In our study, we observed upregulation of DEGs related to three ERF1B-like (Soltu.DM.09G026520.1, Soltu.DM.11G001310.1, and Soltu.DM.05G020900.1) from MAPK signaling pathway and one ERF096-like (Soltu.DM.09G026 500.1) from plant-pathogen interaction pathway (**Figures 4C, D**). Further investigation is needed to understand how Pi07586 regulates the ERF1B-like and ERF096-like in the plant immune system.

Pathogenesis-related (PR) proteins play a crucial role in plants’ innate immune response to biotic and abiotic stress (Zribi et al., 2021). For instance, Co-expression of *AP24* and *PR2* in the chloroplast enhanced resistance against filamentous pathogens (Boccardo et al., 2019). The anti-oomycete activity of PR1 proteins from tomato and tobacco exhibits fungicidal activity against *P. infestans in vitro* and *in vivo* (Niderman et al., 1995). The antibacterial mechanism of PR1 involves cross-border transport, targeting the subunits of AMPK kinase complexes in *P. infestans*, inhibiting their phosphorylation activity, and suppressing the growth and infection of *P. infestans* (Luo et al., 2023). In our study, the three downregulation of *PR1C-like* (Soltu.DM.10G014410.1, Soltu.DM.10G014420.1, and Soltu.DM.10G014400.1) in *Pi07586* transgenic lines (**Figure 4D**). The results indicate that Pi07586 partially suppresses plant immunity by manipulating the expression of PR genes. Chloroplast photosystem centers serve as a potential source of rapidly generated ROS for defense against biotic attacks. Biotic attacks universally downregulate photosynthesis-related gene expression (Berger et al., 2004; Zou et al., 2005). Notably, photosynthetic antenna protein DEGs were significantly downregulated in Pi07586 transgenic plants (**Table 2**, **Figure 4F**). This suggests that Pi07586 can modulate plant immunity by impacting photosynthesis.

Recent research has shown that phytohormone SA, JA, and ET are responsible for primary defense, while plant growth regulators, such as auxins, cytokinins, ABA, and gibberellins, also contribute to plant immunity. Hormonal signaling pathways are often interconnected, and this can lead to synergistic or antagonistic functions, such as the antagonism between SA and JA pathway, the synergism between JA and ET pathways (Han and Kahmann, 2019; Huang et al., 2020). Although the antagonism between SA and JA pathway, all of them can enhance late blight resistance in potatoes (Halim et al., 2007; Luo et al., 2021; Yang et al., 2022). Our data showed that the JA, JA-Ile, and ABA are significantly lower levels in Pi07586 transgenic plants, while IAA and SA level changes remained nonsignificant (**Figure 6**). A mark gene *MYC2* (Soltu.DM.06G022820.1) of the JA pathway was downregulated expression in Pi07586 transgenic lines (**Figure 4E**). This suggested that Pi07586 may inhibit transgenic plant immunity by disturbing JAs’ biosynthesis and signal transduction. ABA has a negative role in plant resistance against some biotrophic flamentous pathogens, such as *Fusarium graminearum*, *M. oryzae* (Jiang et al., 2010; Buhrow et al., 2016), while plants require the ABA pathway for resistance against several necrotrophic pathogens, including *Pythium irregulare*, *C. miyabeanus* (Adie et al., 2007; De Vleesschauwer et al., 2010). Consistent with previous studies, exogenous ABA application make potato more susceptible to *P. infestans* at the early stage of infection (Liu et al., 2020). As *P. infestans* is a hemibiotrophic pathogen, which employs a biphasic infection strategy during early infection and then changes to a necrotroph when the host plant cells have been destroyed (Rodenburg et al., 2019). Therefore, we supposed that Pi07586 reduces plant immunity via inhibiting ABA biosynthesis, which involves plant resistance against *P. infestans* at the stage of necrotroph. Finally, although not all of the selected DEGs were directly involved in late blight resistance, the present results confirmed that Pi07586 suppresses host resistance, at least in part, by altering the expression of defense-related genes, and photosynthetic antenna protein genes, and the content of JA, JA-Ile, and ABA.

## 5 Conclusions

In this study, we identified and characterized an early expressed nuclear effector Pi07586 in potatoes. Transgenic potato plants overexpressing *Pi07586* demonstrated increased sensitivity to *P. infestans* compared to wild-type plants. Through RNA-seq analysis of transgenic and wild-type plants inoculated with *P. infestans*, we identified 793 differentially expressed genes, including 595 upregulated genes and 198 downregulated genes. Furthermore, the differences in hormone content, specifically ABA, JA, and JA-Ile, indicate a potential association between the virulence function of Pi07586 and these hormones. We conclude that Pi07586 acts as a virulence factor in the interaction of potato and *P. infestans*, providing a new example of a non-RxLR/CRN cytoplasmic effector.

## Data availability statement

The datasets presented in this study can be found in online repositories. The names of the repository/repositories and accession number(s) can be found below: https://www.ncbi.nlm.nih.gov/, PRJNA993004.

## Author contributions

YX: Writing – review & editing, Investigation, Writing – original draft. DZ: Investigation, Writing – original draft, Methodology. SC: Writing – original draft, Formal Analysis. LY: Formal Analysis, Writing – original draft, Resources. DeZ: Resources, Writing – original draft. HW: Funding acquisition, Supervision, Writing – review & editing.
