# Supplementary material for: Deciphering the underlying immune network of the potato defense response inhibition by Phytophthora infestans nuclear effector Pi07586 through transcriptome analysis
Source: Front Plant Sci. 2023 Sep 22;14:1269959. doi: 10.3389/fpls.2023.1269959 (PMC10556245; doi:10.3389/fpls.2023.1269959)
Supplement: Supplementary file 1 [file DataSheet_1.docx]

Supplementary Material

Article Title

First Author*, Co-Author, Co-Author

Yumeng Xiong*, Di Zhao, Shengnan Chen, Lan Yuan, Die Zhang

*** Correspondence:** Hongyang Wang: [hongyang8318@ynnu.edu.cn](mailto:hongyang8318@ynnu.edu.cn)

# Supplementary Figures and Tables

## Supplementary Figures


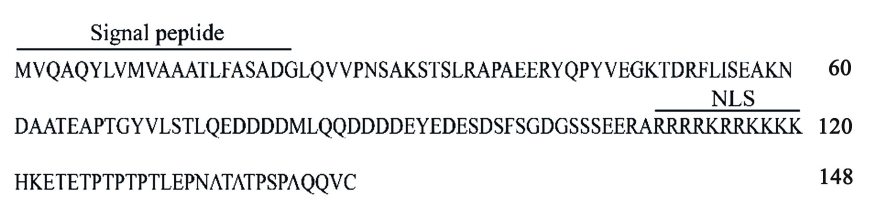


**Supplementary Figure 1.** The amino acid sequence of pi07586. SignalP and HMMER software predicted that the amino acids at the 1–21 positions of the N-terminal end of the protein were signal peptide sequences; Wolfpsort software predicted that the protein was nuclear localized or nuclear-cytoplasmic localized; NLS mapper software predicted that the amino acids at the 109–120 positions (RRRRKRRKKKK) were the cell's approved signal sequence.


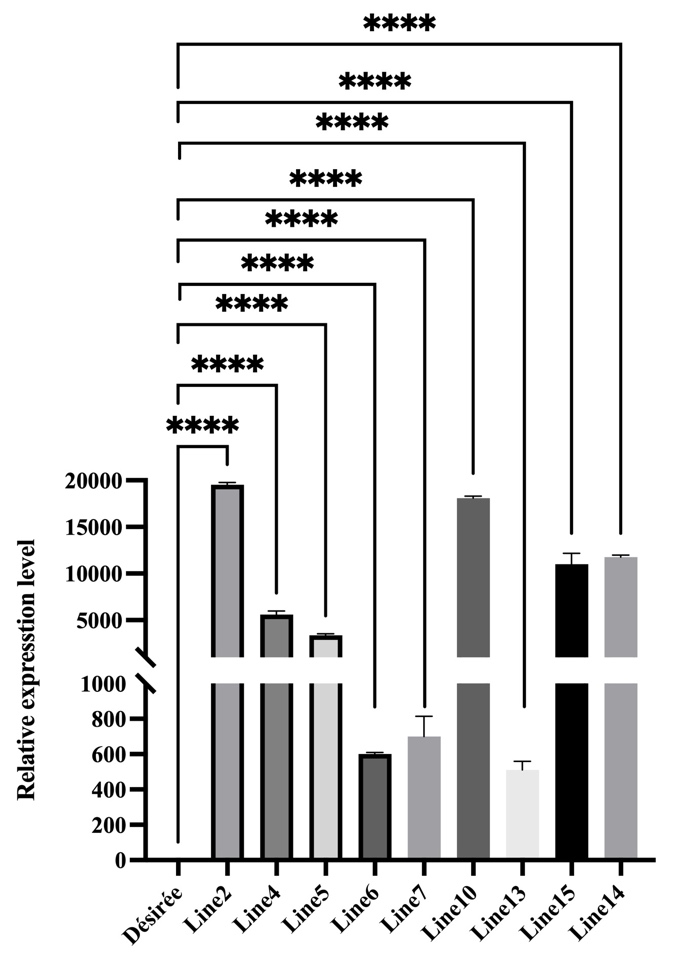


**Supplementary Figure 2.** Relative expression of *Pi07586* in the Désirée and *Pi07586* transgenic potato lines. The internal reference gene is *StEF1α*. The error bars indicate means ± SDs from three independent experiments. One-way ANOVA, ****, p < 0.00001.


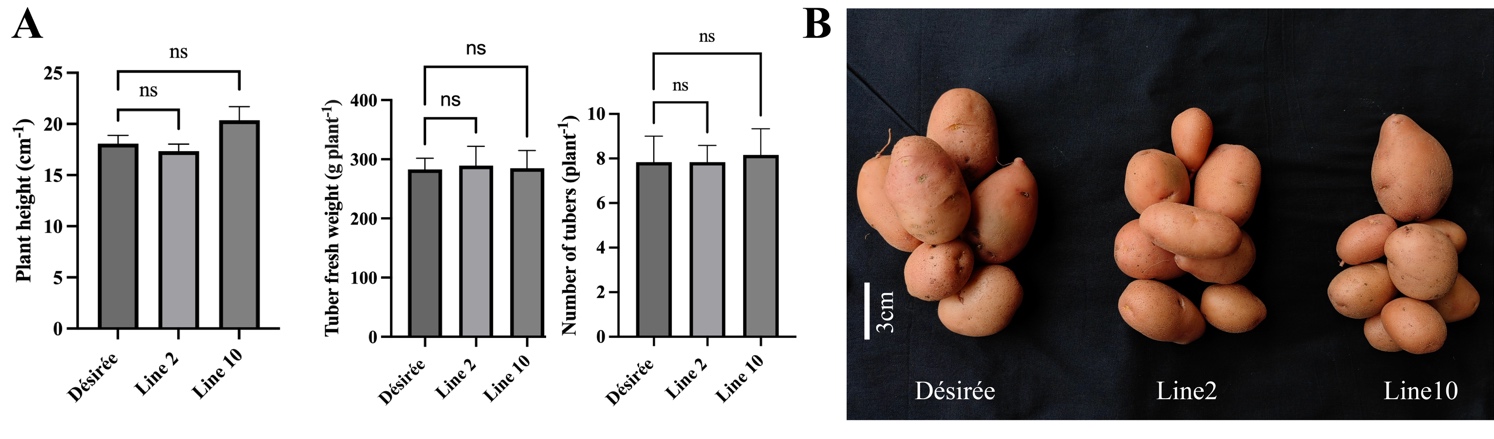


**Supplementary Figure 3.** The height and yield of Désirée and transgenic lines. A. The height, tubers' number, and weight of Désirée, line 2, and line 10. Their heights were measured (after the sterile seedling under grown for about three weeks). Data in the figures are means of three repeats (*n* = 36) of just one harvest of potato plants ± standard deviation (SD).  One-way ANOVA, ns represents Not Statistically Significant. B. The tubers of Désirée, line 2, and line 10. Scale bars indicate 3 cm.
